# Assessment of Retinal Nerve Fiber Layer Changes by Cirrus High-definition Optical Coherence Tomography in Myopia

**DOI:** 10.5005/jp-journals-10028-1223

**Published:** 2017-08-05

**Authors:** Divya Singh, Sanjay K Mishra, Esha Agarwal, Reetika Sharma, Shibal Bhartiya, Tanuj Dada

**Affiliations:** 1 Senior Resident,Department of Ophthalmology, Dr Rajendra Prasad Centre for Ophthalmic Sciences, All India Institute of Medical Sciences New Delhi India; 2 Associate Professor, Department of Ophthalmology, Army Research and Referral Hospital, New Delhi, India; 3 Senior Resident,Department of Ophthalmology, Dr Rajendra Prasad Centre for Ophthalmic Sciences, All India Institute of Medical Sciences New Delhi India; 4 Resident, Department of Ophthalmology, Dr Rajendra Prasad Centre for Ophthalmic Sciences, All India Institute of Medical Sciences New Delhi India; 5 Senior Consultant, Department of Ophthalmology, Fortis Memorial Research Institute, Gurgaon, Haryana, India; 6 Professor, Department of Ophthalmology, Dr Rajendra Prasad Centre for Ophthalmic Sciences, All India Institute of Medical Sciences New Delhi India

**Keywords:** Emmetropia, Retinal Nerve Fiber Layer, Spearman’s rho.

## Abstract

**Introduction:**

To evaluate the relationship between retinal nerve fiber layer (RNFL) thickness measured by Cirrus high-definition (HD) optical coherence tomography (OCT) and the axial length and refractive error of the eye.

**Materials and methods:**

A total of 100 eyes of 100 healthy subjects (age 20-34 years with M/F ratio of 57/43), comprising 50 eyes with emmetropia [spherical equivalent (SE) 0 D], 25 eyes with moderate myopia (SE between -4 D and -8 D), and 25 eyes with high myopia (SE between -8 D and -12 D) were analyzed in this cross-sectional study. Average and mean clock hour RNFL thicknesses were measured by cirrus HD-OCT and compared between the three groups. Associations between RNFL measurements and axial length and SE were evaluated by linear regression analysis.

**Results:**

The average RNFL measurements were significantly lower in high myopia (78.68 +/- 5.67) and moderate myopia (83.76 +/- 3.44) group compared with emmetropia group (91.26 +/- 2.99), also in the superior and inferior mean clock hours. Significant correlations were evident between RNFL measurements and the SE and axial length. The average RNFL thickness decreased with increasing axial length (r = -0.8115) and negative refractive power (r = 0.8397). Myopia also affected the RNFL thickness distribution. As the axial length increased and the SE decreased, the thickness of the superior, inferior, and nasal peripapillary RNFL decreased.

**Conclusion:**

The axial length/refractive error of the eye affected the average RNFL thickness and the RNFL thickness distribution. Analysis of RNFL thickness in the evaluation of glaucoma should always be interpreted with reference to the refractive status. When interpreting the RNFL thickness of highly myopic patients by OCT, careful attention must be given to the inherently thinner RNFL to avoid a false diagnosis of glaucoma.

**How to cite this article:**

Singh D, Mishra SK, Agarwal E, Sharma R, Bhartiya S, Dada T. Assessment of Retinal Nerve Fiber Layer Changes by Cirrus High-definition Optical Coherence Tomography in Myopia. J Curr Glaucoma Pract 2017;11(2):52-57.

## INTRODUCTION

Myopia is a known risk factor for glaucoma. The risk of developing glaucoma is reported to be two to three times higher in myopic individuals than in nonmyopic individuals.^1,2^ These myopic individuals often have enlarged optic disks with more oval configuration and large areas of peripapillary atrophy making diagnosis and management of glaucoma difficult in these cases.^3^

Retinal nerve fiber layer (RNFL) thickness is now considered as one of the sensitive indicator for predicting early glaucomatous damage.^4-6^ Optical coherence tomography (OCT) because of its excellent ability to assess peripapillary RNFL thickness has been extensively used for the diagnosis and follow-up of glaucoma and other optic neuropathies. Cirrus high-definition (HD) OCT (Carl Zeiss Meditec Inc., Dublin, CA), which is one of the newest versions of OCT, provides higher resolution and faster scan rate and has progressively replaced time domain OCT in the evaluation of glaucoma.^7^

The relationship between peripapillary RNFL thickness and myopia has been extensively studied by various investigators with variable results.^8-17^ The purpose of this study was to evaluate the relationship between RNFL thickness measured by cirrus HD-OCT and the refractive error and axial length of the eye.

## MATERIALS AND METHODS

This was a cross-sectional study, carried out after approval by All India Institute of Medical Sciences Ethics Committee and it adhered to the tenets of the Declaration of Helsinki. Informed written consent was taken from each enrolled patient. A total of 100 eyes of 100 healthy young patients were evaluated in the study and patients were divided in three groups according to their refractive status: Group I had 50 patients with emmetropia [spherical equivalent (SE) 0 D], group II had 25 patients with moderate myopia (SE between -4 D and -8 D), and group III had 25 patients with high myopia (SE between -8 D and -12 D). Patients with intraocular pressure (IOP) > 21 mm Hg, glaucoma, cataract, best corrected visual acuity < 20/20, refractive surgery, intraocular surgery, or any ocular pathology other than refractive error, diabetes, neurological disease, or any systemic illness were excluded from the study.

All subjects underwent a comprehensive ophthalmic evaluation which included visual acuity measurement by Snellen’s chart, slit lamp examination, Goldmann applanation tonometry, gonioscopy, perimetry, fundus examination by slit lamp biomicroscopy, and indirect ophthalmoscopy. The refractive error from the manifest refraction was adjusted to the SE. Axial lengths were measured using ocular biometer (IOL Master, Carl Zeiss Meditec Inc.). Peripapillary RNFL thickness was measured using cirrus HD-OCT. Before testing, the pupil of each subject was dilated by instillation of 1% tropicamide and 2.5% phenylephrine hydrochloride to the eye three times over a 10 minutes period. After the subject was seated and aligned properly, three 200x 200-cube optic disk scans were obtained per eye, scans with signal strength below 7 were discarded, and new one was obtained. The scan that had the highest signal strength and was obtained with the least eye movement was selected. All scans were performed by a single operator. The average RNFL thickness, the average thickness in the four sectors — superior, inferior, temporal and nasal. and the average thickness in all clock hour positions was recorded. The superior clock hour was 12 o’clock, and the others were assigned accordingly in a clockwise manner in the right eye and counterclockwise in the left.

### Statistical Analysis

Statistically analysis was done using Statistical Package for the Social Sciences version 16. The Pearson correlation coefficient was used to determine relationship of RNFL thickness with axial length and age. Spearman’s rho was used to evaluate relation between RNFL thickness and SE. One-way analysis of variance was used to compare the other variables among the three groups. Levene’s test was used to assess the homogeneity of the variances. A value of p < 0.05 was considered to be statistically significant.

## RESULTS

A total 100 eyes of 100 patients were evaluated in the study. The patients, as stated above, were divided into three groups on the contrary refractive status, emme-tropic (n = 50), moderately myopic (n = 25) and highly myopic (n = 25). There were total 57 male and 43 female patients. The mean age of patients was 25.57 ± 4.13 (20-34) years. There was no statistically significant difference in the age in three groups (Table 1). Mean SE of the patient was -4.21 ± 4.30 D. Mean SE in group I was -0.37 ± 0.54 D, in group II was -5.64 ± 0.96 D, and in group III was -10.44 ± 1.25 D. Mean axial length of the patient was 24.07 ± 1.44 mm. Mean axial length in group I was 22.89 ± 0.57 mm (22-24 mm), in group II was 24.42 ± 0.34 mm (24-25 mm), and in group III was 26.10 ± 0.64 mm (25-27.11 mm). Mean IOP was 14.70 ± 1.74 mm Hg and mean cup disk ratio was 0.34 ± 0.05. Average RNFL thickness was 86.24 ± 6.62 μm. Average RNFL thickness in group I was 91.26 ± 2.99 μm, in group II was 83.76 ± 3.44 μm, and in group III was 78.68 ± 5.67 μm. Average RNFL thickness was found significantly less in moderate and high myopes (groups II and III) as compared to emmetropes (group I). Similarly, RNFL thickness in superior quadrant and inferior quadrant is significantly low in moderate and high myopes (groups II and III) than emmetopes (group I). Significant difference was also seen in average RNFL thickness and RNFL thickness in superior and inferior quadrant in between moderate and high myopes. The RNFL thickness in nasal quadrant was found significantly less in groups II and III when compared with group I, however, there was no significant difference seen in between groups II and III. Significantly increased RNFL thickness was seen in temporal quadrant in high myopes (group III) than emmetropes and moderate myopes (Table 2).

**Table 1:** **Table 1:** Age distribution of patients in each group

*Group*		*Mean ± SD (years)*	
Group I SE = 0 D, n = 50		26.52 ± 4.33	
Group II -4 D < SE < -8 D, n = 25		24.60 ± 3.57	
Group III -8 D < SE < -12 D, n = 25		24.64 ± 3.98	
Total		25.57 ± 4.13	

p = 0.0705

**Graphs 1A and B:**
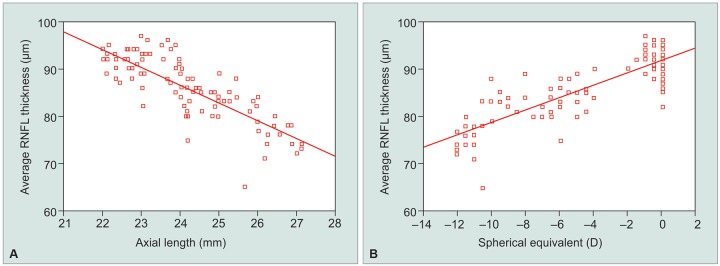
Scatter plots of the average RNFL thickness against: (A) Axial length; (B) spherical equivalent

**Graphs 2A to D:**
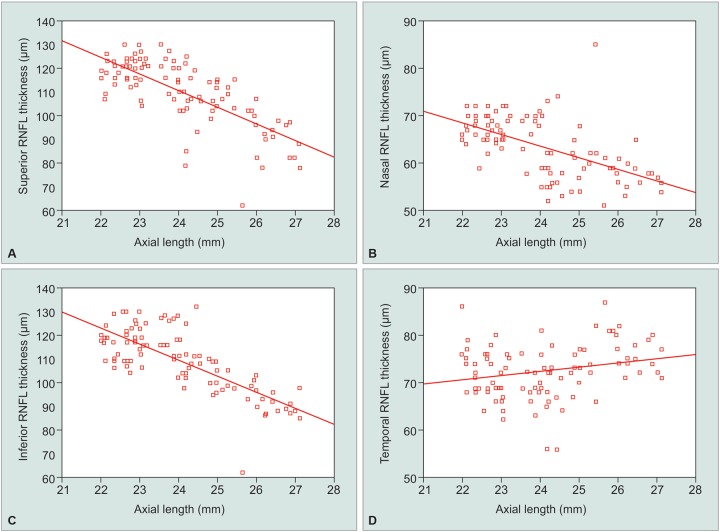
Scatter plots of the RNFL thickness in each quadrant against the axial length

No significant correlation was seen between RNFL thickness and age (p-value = 0.05). Significant correlations were seen between RNFL measurements and the SE and axial length. The average RNFL thickness decreased with increasing axial length (r = -0.8115) and negative refractive power (r = 0.780) (Graph 1). The RNFL thickness in different quadrants were also seen significantly associated with SE and the axial length (Table 3). As the axial length increased and the SE decreased, the thickness of the superior, inferior, and nasal peripapillary RNFL decreased (Graph 2). The RNFL thickness in temporal quadrant shows a week positive correlation with negative SE and positive insignificant correlation with axial length. Overall, RNFL thickness decreased by 3.74 pm/mm increase in axial length and 1.30 μm for every 1 diopter sphere.

**Table 2:** **Table 2:** RNFL thickness measurements (in |jm) of subgroups classified based on spherical equivalent

*RNFL thickness (μm)*		*Group I SE* *= 0 D n = 50*		*Group II -4 D < SE* *< -8 D, n = 25*		*Group III -8 D < SE* *< -12 D, n = 25*		*Groups I and* *II p-value*		*Groups II and* *III p-value*		*Groups I and* *III p-value*	
Average		91.26 ± 2.99		83.76 ± 3.44		78.68 ± 5.67		0.001		0.001		0.001	
Superior		119.02 ± 6.34		107.16 ± 10.76		95.12 ± 12.99		0.001		0.001		0.001	
Nasal		67.3 ± 3.22		59.40 ± 5.83		60.00 ± 6.16		0.001		1.00		0.001	
Temporal		61.32 ± 4.76		60.36 ± 6.36		66.16 ± 4.63		1.00		0.001		0.001	
Inferior		117.94 ± 7.22		107.36 ± 8.59		93.24 ± 9.07		0.001		0.001		0.001	

**Table 3:** **Table 3:** Correlation coefficients between various parameters

*RNFL*		*Axial length*				*Spherical equivalent*			
*thickness*		*Correlation(r)*		*p-value*		*Correlation(r)*		*p-value*	
Average		–0.8115		0.00		0.780		0.00	
Superior		–0.7322		0.00		0.708		0.00	
Inferior		–0.7496		0.00		0.767		0.00	
Nasal		–0.5606		0.00		0.577		0.00	
Temporal		0.2284		0.02		–0.345		0.00	

## DISCUSSION

Analysis of our results demonstrated average RNFL thickness and RNFL thickness in superior and inferior quadrant to be significantly less in high and moderate myopes when compared to emmetropes. Also, significant difference was seen in these parameters between high and moderate myopes. Similarly, Leung et al^8^ using Stratus OCT found average RNFL thickness and thickness in 12, 1, and 7 o’clock less in high myopes (SE < -6 D) as compared to low to moderate myopes (SE between -6 D and -0.5 D). Kamath and Dudeja,^9^ Wang et al,^10^ and Kang et al^11^ used SD-OCT and found average RNFL thickness and thickness in superior and inferior quadrant to be significantly less in myopes similar to our study. However, contrary results were seen in various studies on comparing RNFL thickness in nasal and temporal quadrant. In our study, RNFL thickness in nasal quadrant was significantly less in moderate and high myopes when compared with emmetropes, whereas increased RNFL thickness was seen in temporal quadrant in high myopes. Kamath and Dudeja,^9^ found significant decrease in RNFL thickness in nasal quadrant and insignificant decrease in temporal quadrant in high myopes whereas Wang et al^10^ and Kang et al^11^ in their study found insignificant change in nasal quadrant and thickening in temporal quadrant. Oner et al,^12^ used RTVue SD-OCT found significant decrease in RNFL thickness in all quadrants except nasal quadrant.

In our study, no significant correlation was seen in between age and RNFL thickness. Similarly, Leung et al^8^ and Rauscher et al^13^ did not find a significant correlation between age and RNFL thickness. This may be due to relatively young age and narrow age ranges in these and our study. The study by Budenz et al^14^ which included patients with wider age range (18-70 years) found a small but significant correlation with age (2 microns of RNFL thinning per decade).

In our study, the average RNFL thickness decreased with increasing axial length (r = -0.8115) and negative refractive power (r = 0.780). Similar results were seen in various other study. Budenz et al^14^ noted a significant decrease in RNFL thickness with increasing axial length, equal to 2.2 microns/mm. In our study, RNFL thickness decreased by 3.74 μm/mm increase in axial length and 1.30 μm for every 1 diopter sphere increase in negative SE. In a study by Kang et al,^11^ regression coefficients determined by linear regression analysis for the SE and axial length were 1.310 μm/D and -2.205 μm/mm respectively.

In a study by Rauscher et al,^13^ RNFL thickness decreased 7 microns for every 1 mm increase of axial length and 3 microns for every 1 diopter sphere increase of myopia which was more when compared to our study and previous other study. This decrease in RNFL with increasing Axial length can be because of larger AL which causes larger surface area and distribution of an equal amount of retinal nerves in larger area causing thinner RNFL. However, Hoh et al^15^ and various other studies^18,19^ failed to show any statistically significant correlation between RNFL thickness and axial length or SE. These differences can be due to poorer resolution of earlier generation OCT used in these studies. The cirrus HD-OCT used in our study has a better resolution (5 μ) and a faster scan rate of 26,000 A scans/second.

**Graphs 3A to D:**
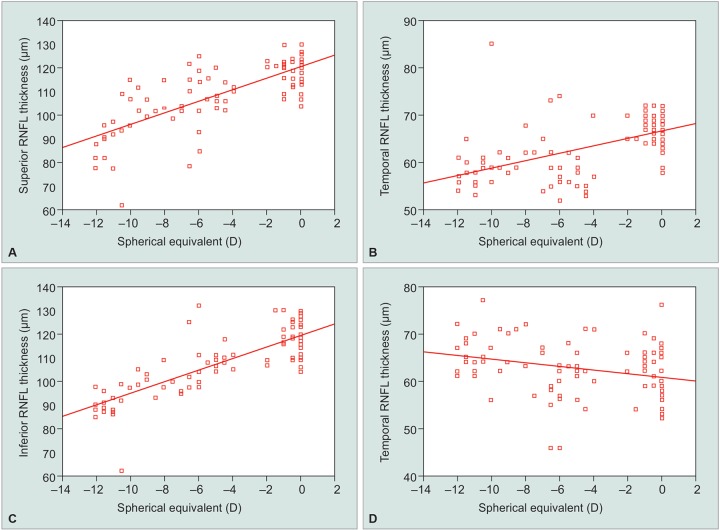
Scatter plots of the RNFL thickness in each quadrant against the spherical equivalent

Quadrant-wise correlation of RNFL thickness with SE and axial length was done in our and various other study to determine if there is any change in distribution pattern of RNFL in myopes (Graph 3). Oner et al,^12^ noted a significant negative correlations between axial length and RNFL thickness in each of the four quadrants in their study, however, correction of magnification effect by Littmann’s formula eliminated the relationship between RNFL thickness and axial length in each sector in their study. Several other studies in the past have also used magnification factor, as it has been found that actual scanning radius in eyes with greater axial length (myopic eyes) could be longer than 1.73 mm due to magnification effect, so using same-sized scan circle for measuring RNFL in patients with different degrees of myopia might be misleading because the RNFL thickness decreases with increasing distances from the optic disk. Although the actual scanning radius is longer than 1.7 mm in myopes, it necessarily does not mean that the RNFL is being measured farther from the disk margin as the optic disk size also increases with myopia, so we did not correct for magnification factor in our present study as there are chances of overestimation of RNFL after correction of magnification factor.

In our study, we found that as the axial length increased and the SE decreased, the thickness of the superior, inferior, and nasal peripapillary RNFL decreased, however, RNFL thickness in the temporal quadrant showed a week positive correlation with negative SE and positive insignificant correlation with axial length. Rauscher et al^13^ in their study found RNFL thickness in superior and inferior quadrant to decrease with increasing axial length and higher SE whereas nasal and temporal RNFL thickness showed no association with myopia. Leung et al^8^ reported significant positive correlation between RNFL thickness and axial length and negative correlation with SE in all clock hours except temporal in their study.

The main limitation of our study was that we did not correct for magnification factor and have not included hypermetropes in our study.

## CONCLUSION

The axial length and refractive error of the eye affect the average RNFL thickness and the RNFL thickness distribution, so the analysis of RNFL thickness in the evaluation of glaucoma should always be interpreted with reference to the refractive status of the patient.
